# The prevalence of the metabolically healthy obese phenotype in an aging population and its association with subclinical cardiovascular disease: The Brazilian study on healthy aging

**DOI:** 10.1186/1758-5996-6-121

**Published:** 2014-11-07

**Authors:** Lara Roberson, Sameer Shaharyar, Ehimen Aneni, Wladimir Freitas, Michael Blaha, Arthur Agatston, Roger Blumenthal, Raul D Santos, Hamid Feiz, Khurram Nasir, Andrei Sposito

**Affiliations:** Center for Prevention and Wellness Research, Baptist Health Medical Group, Miami Beach, FL USA; Aventura Hospital & Medical Center, Aventura, FL USA; Cardiology Department, State University of Campinas School of Medicine Campinas, Sao Paulo, Brazil; Johns Hopkins Hospital Ciccarone Preventive Cardiology Center, Baltimore, MD USA; Lipid Clinic- Heart Institute (InCor), University of São Paulo Medical School Hospital, Sao Paulo, Brazil; Department of Epidemiology, Robert Stempel College of Public Health, Florida International University, Miami, FL USA; The Johns Hopkins Ciccarone Center for the Prevention of Heart Disease, Baltimore, MD USA; Department of Medicine, Herbert Wertheim College of Medicine, Florida International University, Miami, FL USA; Cardiology Division, Faculty of Medical Sciences, State University of Campinas (UNICAMP), Cidade Universitária, Campinas, SP 13084-971 Brazil

**Keywords:** Obesity, Aging, Metabolic Syndrome, Subclinical CVD

## Abstract

**Background:**

Current literature has elucidated a new phenotype, metabolically healthy obese (MHO), with risks of cardiovascular disease similar to that of normal weight individuals. Few studies have examined the MHO phenotype in an aging population, especially in association with subclinical CVD.

**Research design and methods:**

This cross sectional study population consisted of 208 octogenarians and older. Anthropometrics, biochemical, and radiological parameters were measured to assess obesity, metabolic health (assessed by the National Cholesterol Education Program –Adult Treatment Panel (NCEP-ATP III) criteria), and subclinical measures of CVD.

**Results:**

The prevalence of MHO was 13.5% (N = 28). No significant association with MHO was noted for age, coronary artery calcium score, cIMT, or hs-CRP > 3 mg/dl (p = NS).

**Conclusions:**

Our results suggest that the MHO phenotype exists in the elderly; however, subclinical CVD measures were not different in sub-group analysis suggesting traditional metabolic risk factor algorithms may not be accurate in the very elderly.

## Introduction

Obesity in the elderly is rising as obese and overweight adults continue to reach old age; however, clinically, it retains a controversial status due to the “obesity paradox”--a term referring to increased survival rates of overweight elderly from cardiovascular diseases as compared to their normal weight counterparts [1]. This is complicated by the fact that the older overweight and obese individuals are at greater risk of developing these same diseases, often because of the close ties between obesity and metabolic syndrome (MetS). Within the obese population, the small portion who do not display the typical associated metabolic disorders are termed “metabolically healthy obese” (MHO) [2]. Research has yielded mixed results on the true normal weight counterparts and others showing increased risks [3–6]. There are few studies, however, which have examined the prevalence of “MHO” in the very aged (≥80) population [7–9]. In this study, we examined the MHO phenotype a primarily octogenarian cohort and its association with subclinical cardiovascular disease and inflammation.

## Research design and methods

### Subjects

This cross sectional study was conducted at the Biocardios Institute of Cardiology, Brasilia, Brazil. To participate, subjects had to be greater than 80 years of age and free of clinical evidence of cardiovascular disease. All participants underwent a detailed clinical examination along with quality of life assessment including self-reported physical activity levels.

### Anthropometric & lifestyle assessment

Weight and height were measured to the nearest 0.1 kg and 0.1 cm respectively, using standardized equipment and procedures and the BMI calculated from these values [10]. BMI was subdivided into “normal weight” if BMI was less than 25, and “overweight/obese” if BMI was 25 or greater. Waist circumference was assessed at minimal inspiration to the nearest 0.1 cm, midway between the last rib and the iliac crest; and abdominal obesity was defined as >88 cm for women, and 102 cm for men. Combining Overweight (25–29.9 kg/m^2^), and Obese (≥30 kg/m^2^) into a single category “overweight/obese” was done to increase statistical power due to the relatively small size of the study cohort. Overweight/obese, therefore, was defined as BMI > = 25 and/or meeting waist circumference criteria for abdominal obesity. Physical activity was self reported and scored “meets recommendations” or “does not meet recommendations” using the World Health Organization’s “Global recommendations on physical activity for health” information sheet [11]. This recommendation suggests adults over the age of 65 do at least 150 minutes of moderate-intensity aerobic physical activity per week or 75 minutes of vigorous-intensity aerobic physical activity or an equivalent combination of moderate- and vigorous-intensity activity.

### Metabolic health & obesity

Details of biochemical collection methods have been published elsewhere [12]. A modified National Cholesterol Education Program- Adult Treatment Panel III (NCEP-ATP III) definition of metabolic syndrome (MetS) criteria was used to define metabolically healthy (<2 risk characteristics) verses unhealthy [13]. Systolic and diastolic blood pressure, fasting blood glucose (FBG), HDL-C, and Triglycerides were included in the risk profile. Waist circumference was not used in the definition of MetS because it was used as part of the definition of overweight/obesity and it is a better indicator of obesity in the elderly for reasons described in the discussion [14]. Therefore, rather than the traditional <3 risk characteristics cut off to be considered “Metabolically Healthy”, a cut point of <2 risk characteristics was utilized. Blood pressure was considered to be “at risk” if systolic ≥130 mmHg, diastolic ≥85 mmHg, or the subject was on antihypertensive medication. If FBG was ≥110 mg/dL, or the subject was on diabetes medication, then FBG was considered to be “at risk”. If HDL-C was <50 mg/dL in women, <40 mg/dL in men, or the subject was on nicotinic acid, HDL-C was considered “at-risk”. If Triglycerides were ≥150 mg/dL, or the subject was on fibrates, then triglycerides were considered “at-risk”.

### Subclinical disease measures

Multidetector-row cardiac CT for coronary artery calcium score (CACS) was used to detect subclinical atherosclerosis. Those with a CACS = 0 were considered “normal”, which is associated with a low risk of CVD events in literature [15]. CACS >0 were subdivided into 3 groups (0–100;100–400 and >400) in an attempt to determine differences in scores across groups. Carotid sonography was used to examine intima-media thickness (CIMT) and was analyzed as a continuous variable. High-sensitivity C reactive protein (hs-CRP) and uric acid was measured to assess degree of underlying inflammation and endothelial function.

### Statistical analysis

The analyses compared the four metabolic and weight phenotypes and measures of demographics, subclinical disease, and metabolic risks using chi-square for categorical variables and ANOVA for normally distributed continuous variables. Ten year Framingham risk scores were calculated as per published literature [16]. Pair wise comparisons were tested when significant differences were found. For skewed continuous data, the Kruskal-Wallis test was used. A p-value <0.05 was considered statistically significant.

## Results

### MHO and Subclinical CVD

The cross sectional study population consisted of 208 individuals (79% Female), age 80 and older (mean age 84 ± 4, range 80–102). Overall, 9.0% of men and 14.6% of women were considered to be MHO. The most common metabolic syndrome components were abnormal blood pressure, followed by high waist circumference, and low HDL. When analyzed by four categories inclusive of metabolic clustering and weight, there were no significant differences between metabolically healthy, normal weight (MHNW); metabolically unhealthy, normal weight (MUNW); MHO; and metabolically unhealthy obese/overweight (MUHO) in most subclinical measures of CVD, inflammation, and age (Table 1). Only median Uric Acid was significantly different across the four groups; however, when it was categorized into clinically relevant categories (>6 mg/dL or ≤6 mg/dL), these differences became null due to small sample size. When CACS was categorized into 0, 1–100, 100–400, ≥400 no relationships were found with metabolic status or obesity. A total of 21 (11.9%) adults were found to have calcium scores of 0.

**Table 1:** **Metabolic Health, Obesity Status, and Subclinical Disease in the study population**

	Overall N = 208	1. Metabolically healthy normal weight (N = 21; 10.1%)	2. Metabolically unhealthy normal weight (N = 43; 20.7%)	3. Metabolically healthy overweight/obese (N = 28; 13.5%)	4. Metabolically unhealthy overweight/obese (N = 116; 55.8%)	Overall ***p***-values
Age, yrs (median, IQR)	83 (81,87)	85 (82,87)	83 (81,87)	84 (81,89)	83 (81,87)	0.55
Gender (Female), n (%)	164 (79)	13 (62)^c^	29 (67)^e^	24 (86)	98 (84)^c,e^	0.02
BMI, kg/m2(median, IQR)	26 (23,29)	22 (20,23)^b,c^	23 (21,24)^d,e^	26 (24,30)^b,d^	28 (26,31)^c,e^	0.000
Current Smokers, n (%)	5 (2)	0 (0)	1 (2)	1 (4)	3 (3)	0.87
Physical Activity Reaches WHO recommendation, n (%)	54 (27)	8 (40)	13 (32)	8 (29)	25 (22)	0.33
Systolic Blood Pressure,mmHg (median, IQR)	142 (132,57)	139 (119,145)	140 (129,165)	136 (131,151)	143 (135,157)	0.12
Diastolic Blood Pressure, mmHg (median, IQR)	73 (67,82)	74 (66,74)	71 (67,80)	73 (67,83)	75 (68,84)	0.72
“At Risk” Blood Pressure, n (%)	129 (62)	11 (52)^a,b,c^	38 (88)^a,e^	25 (89)^b,f^	115 (99)^c,e,f^	0.000
HbA1C (median, IQR)	6.0 (5.7,6.3)	5.8 (5.6,6.1)	5.8 (5.6,6.3)	5.7 (5.5,6.1)	6.1 (5.8,6.5)	0.02
Fasting Blood Glucose, mg/dL (median, IQR)	95 (89,105)	93 (86,96)	95 (89,107)	91 (85,93)	99 (89,113)	0.03
“At Risk” Fasting Blood Glucose, n (%)	40 (25)	3 (10)^a,c^	11 (34)^a,d^	0 (0)^d,f^	28 (36)^c,f^	0.000
Total Cholesterol, mg/dL (mean, SD)	198 (41)	220 (36)	190 (40)	207 (29)	196 (44)	0.03
LDL Cholesterol, mg/dL (mean, SD)	114 (36)	132 (33)	108 (37)	117 (23)	112 (38)	0.09
HDL Cholesterol mg/dL (median, IQR)	54 (45,63)	62 (56,70)^c^	55 (45,63)	60 (54,67)^f^	49 (42,60)^c,f^	0.000
“At Risk” HDL, n (%)	65 (31)	0 (0)^a,c^	14 (33)^a,d^	0 (0)^d^	51 (44)^c^	0.000
Triglycerides, mg/dl(median, IQR)	115 (90,156)	104 (91,119)	105 (88,152)	100 (83,113)^f^	134 (99,167)^f^	0.000
“At Risk” Triglycerides, n (%)	123 (59.4)	2 (10)^a,c^	30 (70)^a,d^	1 (4)^d,f^	90 (78)^c,f^	0.000
Waist Circumference, cm (mean, SD)	94.22 (12.1)	80.4 (7.3)^b,c^	83.9 (8.5)^d,e^	95.8 (10.0)^b,d^	100.1 (9.6)^c,e^	0.000
“At Risk” Waist Circumference, n (%)	129 (62.3)	0 (0)^b,c^	0 (0)^d,e^	23 (82.1)^b,d^	106 (91.4)^e,c^	0.000
Framingham Risk Score (median, IQR)	31 (20,48)	27 (21,37)	32 (20,51)	23 (17,35)	34 (21,50)	0.02
hs-CRP mg/dL (median, IQR)	1.9 (1.0,3.4)	1.9 (1.0,3.8)	1.5 (1.0,3.3)	1.8 (1.1,2.6)	2.1(1.0,3.9)	0.51
hs-CRP > 3 mg/dL, n (%)	66 (32)	7 (33)	13 (30)	4 (14)	42 (37)	0.15
Uric Acid, mg/dL (median, IQR)	5.1 (4.4,6.0)	4.9 (3.9,5.5)	5.0 (4.3,5.7)	5.7 (5.5,6.1)	5.3 (4.6,6.4)	0.01
Uric Acid >6 mg/dL, n (%)	52 (25)	3 (14)	7 (16)	4 (14)	38 (33)	0.06
Right CIMT, mm (mean, SD)	0.82 (0.15)	0.94 (0.23)	0.79 (0.12)	0.80 (0.09)	0.81 (.15)	0.14
Left CIMT, mm (mean, SD)	0.86 (0.17)	0.91 (0.18)	0.85 (0.14)	0.87 (0.09)	0.84 (0.20)	0.43
CACS, Agatston (median, IQR)	142 (34,424)	176 (73,698)	70 (11,474)	162 (46,403)	172 (36,392)	0.36
CACS = 0, n (%)	21 (12)	2 (10)	3 (8)	3 (12)	13 (14)	0.68
CACS = 1-100, n (%)	57 (32)	5 (26)	18 (46)	9 (34)	25 (27)	
CACS = 100-400, n (%)	53 (30)	6 (32)	8 (20)	7 (27)	32 (34)	
CACS ≥ 400, n (%)	46 (26)	6 (32)	10 (26)	7 (27)	23 (25)	

^a^ = p < 0.05 between groups 1 and 2; ^b^ = p < 0.05 between groups 1 and 3; ^c^ = p < 0.05 between groups 1 and 4; ^d^ = p < 0.05 between groups 2 and 3; ^e^ = p < 0.05 between groups 2 and 4; ^f^ = p < 0.05 between groups 3 and 4.

BMI = body mass index; WHO = world health organization; HbA1C = glycated hemoglobin; CACS = coronary artery calcium score; CIMT = carotid intima media thickness; hs-CRP = high sensitivity c-reactive protein.

## Discussion

In this very elderly Brazilian population, the prevalence of metabolically healthy obesity was 13.5% overall. Our results are somewhat in contrast to those reported by Wildman et al. who examined the prevalence of the MHO phenotype in the NHANES database, including those aged 80 years and above. Among normal weight individuals, 67% of our population was metabolically unhealthy compared with 56% in the NHANES population. Among the obese, 81% of participants were found to be metabolically unhealthy compared to 22% reported by Wildman et al. These differences may be due to the smaller size of our sample, or due to the different criteria for obesity in our study or may represent an ethnic difference in the prevalence of these phenotypes. Further studies are needed in order to determine the true prevalence of these phenotypes in the very elderly population [17].

When we examined our metabolic risk factors independently, we found that high blood pressure was very common (62%), correlating with previous reports indicating rates of 60-80% in the elderly [12]. Low HDL and high triglycerides were present in over half of the population. High fasting plasma glucose was present in just over one quarter. As one can see from the data, risk factors become less useful at predicting events as most elderly people have at least one “at risk” condition. Using these risk factors to stratify this population into higher or lower risk categories therefore becomes much more difficult.

The degree of subclinical CVD assessed by mean calcium score, CIMT, and inflammation were no different among the four metabolic categories. CACS has been shown to be superior to risk factors in predicting CVD risk in literature, both independently and as an addition to risk factor scores [18–21], although no consensus has been reached on the same. Newman et al. studied CACS in older adults and found that hypertension, diabetes, total, LDL, and HDL cholesterol levels were not significantly different across the levels of calcium score [22], suggesting that traditional risk factors may not be associated with subclinical atherosclerosis in the very elderly. Our study is in partial agreement, in that there are significant differences in risk factor prevalence across groups, but these have not translated into a difference in subclinical disease burden across groups. Literature has identified a number of age related changes in biological processes underlying atherogenesis in the elderly, possibly diminishing the utility of traditional factors - which may be responsible for our results [23–26]. Alternatively, it is possible that the markers of subclinical disease used in our study become less useful with advancing age, although we are not aware of any literature documenting the same. Interestingly, 11.9% of our cohort were found to have calcium scores of 0 which has been associated with a very small risk of a CVD event in the literature [15]. This raises the interesting possibility that these individuals with CACS = 0 may represent a subgroup in our population with an extremely low “natural” or “baseline” risk of CVD, deserving of further exploration.

### Limitations

This study was a cross-sectional survey which only examined the subjects at one time point. While this allows us to examine the prevalence of the MHO phenotype in people aged 80 years and above and its association with subclinical CVD, it does not allow us to establish a temporal relationship without follow-up studies. Another limitation of our study was the small sample size. Living into the 8^th^ decade of life is in itself rare, and being free of cardiovascular disease at that time is even more uncommon. Having data on only 208 individuals limited the power to interpret the results of our study. Future, larger studies may bring out differences in subclinical disease across metabolic risk/weight groups which were null in our study and have the power to examine groups in context of increasing number of metabolic risks. This would help identify the most predictive traits among the elderly, and perhaps enable researchers to eliminate extraneous risk factors in this understudied group.

Usefulness of BMI to diagnose obesity in the elderly is poor due to a weight instability and sarcopenia (or muscle wasting) in the very elderly [27]. The elderly population is known to be subject to involuntary weight loss, with as much as 15-20% of the elderly losing 5% of their total body weight over 5–10 years [28]. Additionally, published literature has suggested that different BMI cutoffs may be more appropriate for the very elderly (in which BMI range of 22–29 kg/m^2^ is considered normal) [29]. Using BMI as an indicator of obesity with cutoffs of >25 kg/m^2^ may have caused confounding in our results and blunting of the associations. As people age (especially after the age of 65), muscle tissue is lost and replaced with metabolically active adipose tissue. Sarcopenic obesity has been coined to describe the condition of excess fat without the accompanying increase in extra skeletal muscle to support the weight seen in younger individuals [1]. This makes older people more likely to be considered obese by body fat standards and less likely using traditional BMI.

Our study was also subject to survivorship bias. Subjects over the age of 80 represent a small fraction of their initial birth cohort, meaning they possessed some unique trait giving them a survival advantage over their peers, allowing them to reach the age of 80. This unstudied trait may have protected them from cardiovascular disease in their life course, which would help explain the wide variability in risk profiles. Additionally, most of our population was female (79%), which limits the generalizability of our findings.

## Conclusion

Our study demonstrates significant differences in metabolic risk factor prevalence across obesity phenotypes without corresponding differences in subclinical disease burden or inflammatory activity in the very elderly. When assessing high risk populations such as the very elderly, the usefulness of traditional risk factor assessment (such as Framingham) should be carefully questioned. Most people >80 have at least one metabolic risk factor, which may limit the ability of employing risk factors alone in an attempt to stratify population into different risk categories. Therefore, actual measures of subclinical disease are potentially important as they provide a snapshot of disease progression and more accurate event prediction. However, our study did not document any significant differences in subclinical disease burden across groups. Since our study is a cross-sectional one, it is beyond the scope of the present study to determine if the traditional risk factors or subclinical measures are a correct representation of this population’s risk. This study identified the existence of the MHO phenotype within this population; however, failed to show an association between the metabolic phenotype and the subclinical cardiovascular disease burden despite significant differences in traditional risk factor profiles. Few studies have examined the clinical utility and additional risk prediction of subclinical disease measures in the very elderly, and none in association with metabolic syndrome and obesity. Future long term follow up studies are needed to elucidate the connection between metabolically healthy obesity, the progression of cardiovascular disease, and ultimately, morbidity and mortality in this rapidly expanding population.

### Consent

Written informed consent was obtained from the patients for the publications of this report and any accompanying images.
